# The use of gaze to study cognition: limitations, solutions, and applications to animal welfare

**DOI:** 10.3389/fpsyg.2023.1147278

**Published:** 2023-05-02

**Authors:** Vanessa A. D. Wilson, Emily J. Bethell, Christian Nawroth

**Affiliations:** ^1^Department of Comparative Cognition, Institute of Biology, University of Neuchâtel, Neuchâtel, Switzerland; ^2^Department of Comparative Language Science, University of Zurich, Zurich, Switzerland; ^3^Center for the Interdisciplinary Study of Language Evolution (ISLE), University of Zurich, Zurich, Switzerland; ^4^Research Centre in Evolutionary Anthropology and Palaeoecology, Liverpool John Moores University, Liverpool, United Kingdom; ^5^Institute of Behavioural Physiology, Research Institute for Farm Animal Biology (FBN), Dummerstorf, Germany

**Keywords:** looking time, attention, visual bias, animal behavior, social cognition, methodology, study design, welfare indicators

## Abstract

The study of gaze responses, typically using looking time paradigms, has become a popular approach to improving our understanding of cognitive processes in non-verbal individuals. Our interpretation of data derived from these paradigms, however, is constrained by how we conceptually and methodologically approach these problems. In this perspective paper, we outline the application of gaze studies in comparative cognitive and behavioral research and highlight current limitations in the interpretation of commonly used paradigms. Further, we propose potential solutions, including improvements to current experimental approaches, as well as broad-scale benefits of technology and collaboration. Finally, we outline the potential benefits of studying gaze responses from an animal welfare perspective. We advocate the implementation of these proposals across the field of animal behavior and cognition to aid experimental validity, and further advance our knowledge on a variety of cognitive processes and welfare outcomes.

## Introduction

Eye gaze is an implicit behavior that is used to determine visual attention, and provides a useful proxy measure of cognition, e.g., preferences, comprehension, knowledge, beliefs, and memory, in non-verbal subjects. Consequently, gaze measures such as gaze direction, looking time, anticipatory looking and gaze following, have become established tools in cognitive research (Tafreshi et al., 2014). Gaze measures have advanced our understanding of cognitive processes, but there are notable limitations. Despite calls in developmental psychology that caution about using gaze to understand higher-order cognition (Bogartz et al., 2000; Aslin, 2007; Tafreshi et al., 2014), gaze measures persist in this field, as well as in the study of animal cognition. In this perspective paper, we draw on literature both from developmental and comparative research. We focus on these fields, since methodological improvements with non-verbal subjects could lead to a better understanding of both the ontogenetic and the evolutionary origins of specific cognitive traits Moreover, whilst these fields both seek to answer similar questions regarding cognitive functions, and face similar methodological challenges, we suggest they could benefit from a broader exchange of ideas. Using examples from socio-cognitive studies utilizing gaze measures, we review the existing issues, propose solutions and improvements to existing methodologies, and address how we can give more consideration to the mechanisms that drive context-specific visual attention; we also discuss the general benefits, as well as future implementations, of a gaze-based approach for animal welfare related issues.

## Using gaze to study cognition

### Methods and applications

One of the most common approaches is the preferential looking paradigm–a method that compares length of time that gaze is directed toward different stimuli (images or videos). Stimuli are typically presented simultaneously as a pair, or individually at separate time points. Originally tested on an infant chimpanzee [*Pan troglodytes*, Fantz (1958a)] then with human infants (Fantz, 1958b), the method’s simplicity and adaptability to different environments has made it popular amongst researchers working with animals. While indicating discrimination between stimuli (Koba and Izumi, 2008; Pfefferle et al., 2014), the goal of studies using this paradigm vary, with some aiming to uncover social orientations (Craighero et al., 2011; Franchini et al., 2017) or preferences (Paukner et al., 2017; Adams and Macdonald, 2018), recognition (Fujita, 1987; Schell et al., 2011; Méary et al., 2014; Rakotonirina et al., 2018) as well as memory for stimuli (Gothard et al., 2004; Howard et al., 2018) and shifts in underlying affective states (Bethell et al., 2012). The term *preferential looking* is synonymous with *looking time, attention*, and *gaze.* More recently the terms *visual bias* and *attention bias* have been introduced, most commonly for welfare studies where difference in looking duration to emotionally valenced stimulus pairs is measured (e.g., Crump et al., 2018).

A variation on the preferential looking paradigm is the “violation of expectation” (VoE) paradigm, where visual bias is interpreted as an indicator of surprise in response to something unexpected. VoE paradigms have been used to test expectations about visual perspectives (Arre et al., 2020), physical support violations (Baillargeon and Hanko-Summers, 1990; Cacchione and Krist, 2004; Perez and Feigenson, 2021), object motion (Spelke et al., 1994), and social violations, both in the visual (Rohr et al., 2015; Overduin-de Vries et al., 2016), and auditory domain (Cheney et al., 1995; Bergman et al., 2003; Slocombe et al., 2010).

The habituation/dishabituation paradigm is another adaptation of the preferential looking paradigm. Here, subjects are exposed to a stimulus until they reach a habituation response–measured through a decrease in looking time over multiple exposures–and are then exposed to a different stimulus. Their looking time is compared between the novel stimulus and the last habituation trial (Leslie, 1982). O’Connell and Dunbar (2005) measured perception of causality in chimpanzees using two different conditions. They showed either a probable scene (e.g., hand grasping a banana) followed by an improbable scene (e.g., banana moving on its own) or an improbable followed by probable scene. Chimpanzees looked longer when viewing the improbable clip second (i.e., during dishabituation), but not when viewing the probable clip second, thereby suggesting that the improbable clip produced a causal violation. This approach has been used to study understanding of intention (Dasser et al., 1989) and vocal information (Rendall et al., 1996; Charlton et al., 2012; Baciadonna et al., 2019).

In contrast to the above approaches, anticipatory looking paradigms measure gaze direction prior to the onset of stimuli, providing information about expectations of event outcomes. This approach has been used to test predictions about goal-directed actions (Cannon and Woodward, 2012; Kano and Call, 2014) and related belief systems about object locations (Onishi and Baillargeon, 2005; Southgate et al., 2007; Krupenye et al., 2016).

Finally, gaze following paradigms test the ability to co-orient gaze with another individual (using cues from body, head, or eye orientation). Gaze following is a reflexive response that emerges during infancy (Deaner and Platt, 2003; Shepherd, 2010; Davidson and Clayton, 2016), and has been found in a range of taxonomic groups including reptiles (Wilkinson et al., 2010), birds (Schloegl et al., 2007), and mammals (Davidson and Clayton, 2016). In many birds and mammals, gaze following first emerges in response to the primary carer, suggesting it is a reflexive behavior facilitating behavioral coordination between infant and parent, enhancing learning opportunities (Schloegl et al., 2007; Davidson and Clayton, 2016). In human adults, gaze following is also mediated by multiple social variables (Dalmaso et al., 2020a), suggesting that gaze following is also integrated into less automated pathways for social processing (Shepherd, 2010). Similarly, in other species, evidence is also accumulating for more complex forms of gaze following, such as gaze following around barriers (Zeiträg et al., 2022).

Whilst the above paradigms are well established approaches, utilized both in lab settings and in free-ranging populations of non-human animals, all are inherently prone to conceptual and methodological issues which are often overlooked. In the following section, we outline these limitations and provide potential solutions.

### Limitations to current methods

The primary issue with the above paradigms is in their interpretation of looking behavior. The preferential looking paradigm provides a simple yet effective way to determine discrimination between stimuli. The paradigm was originally developed to test discrimination between perceptual features, but was later adopted as an indicator of expectation or understanding (Tafreshi et al., 2014), and also as a means to assess shifts in underlying emotion (Paul et al., 2005; Bethell et al., 2012). It is through interpretive differences about underlying psychological and physiological states that this method becomes conceptually problematic. That is, preferential looking paradigms only demonstrate that a pair of stimuli can be discriminated, not why the stimuli are discriminated (Aslin, 2007; Tafreshi et al., 2014; Wilson et al., 2021). A study group might look longer at images of out-group members because to different individuals they are novel, threatening or attractive (Fujita, 1987; Méary et al., 2014). Or they might look longer at images of group members because they, again, contain individually-relevant social information (Fujita, 1987; Dufour et al., 2006; Adams and Macdonald, 2018; Rakotonirina et al., 2018). If we don’t know what exactly drives these visual biases, then evaluation in behavioral terms can be precarious and possibly misleading. Context, including the often-ignored factor of individual differences, is everything when it comes to interpretation (Wass, 2014).

From a broader perspective, it is paradoxical that the same response - visual bias - can varyingly be interpreted to indicate preference, interest, novelty, surprise or even levels of anxiety (Bethell et al., 2012; Tafreshi et al., 2014; Wilson et al., 2021); indeed, the use of the phrasing “preferential looking” is in itself misleading, as it imparts an element of choice in responses when they may be purely reflexive (Winters et al., 2015; Wilson et al., 2021). It also implies that visual bias is due to preference rather than avoidance. Such issues have already been raised in the field of infant cognition –Haith (1998) expresses concern for the use of gaze to make interpretations about higher order cognition, with perception-based explanations often being ignored (Bogartz et al., 2000).

A similar case is argued for the VoE paradigm, where a longer gaze is considered to indicate a surprise response. There are two issues here. The first is that, as pointed out above, the “meaning” of a visual bias is determined within the framework of the paradigm, so a longer looking time will only be considered to indicate surprise if a violation of expectation is being used. By comparison, a longer looking time in a different paradigm would not be interpreted as surprise, but as anything ranging from sexual attraction to threat-related attentional capture. Indeed, subjects may just be responding to novelty in the stimuli following familiarization trials (Wang, 2004). The second issue is that it is unclear what exactly should link a “surprise” response to an understanding of the expected outcome, especially in the absence of additional corroborating evidence (Tafreshi et al., 2014).

The habituation-dishabituation paradigm might provide one method to disentangle conflicting interpretations of visual bias. With a habituation phase followed by dishabituation, it is possible that the response to the dishabituation stimulus compared with the habituated one is due simply to the novelty of the new stimulus rather than any meaningful differentiation based on content. One way to deal with this is to add a re-habituation paradigm, where the initial sequence from the habituation trials is re-played after the dishabituation trial (Rendall et al., 1996). If change in response is not due only to change in stimulus, then response to the re-habituated stimulus should reflect the habituated response level. This approach may provide a way to determine whether subjects are simply distinguishing differences in low-level features, or whether they show consistent responses to particular stimulus categories.

Whilst conceptually more stable than the above measures, anticipatory looking is not without its limitations. A popular application of this measure is to predicting actions, and anticipatory gaze has been used to implicitly measure false belief understanding in infants (Southgate et al., 2007), apes (Krupenye et al., 2016), and Japanese macaques (*Macaca fuscata*) (Hayashi et al., 2020). Yet several studies have failed to replicate previous findings that infants implicitly understand false beliefs as measured through anticipatory looking (Dörrenberg et al., 2018; Kampis et al., 2021). This is not to say that predictive gaze is not a reliable measure, rather that it may be dependent on additional factors, such as familiarity of action and agent (Elsner and Adam, 2021) as well as developmental variation, such that predictive gaze might not be a reliable measure for children under the age of 12 months (Daum et al., 2012). Given these complexities, robust interpretations with non-human subjects should rely on careful selection of test stimuli as well as inter-lab replication.

Earlier gaze following studies faced criticism primarily around issues of bias toward primates and anthropocentric interpretation in terms of visual perspective taking and Theory of Mind (van Rooijen, 2010). Subsequently, as research has embraced other taxonomic groups, interpretation has become more nuanced. Currently it is accepted that sensitivity to gaze in conspecifics (or human carers) and gaze following in general, can be innate and reflexive, and show a developmental trajectory (Schloegl et al., 2007; Zeiträg et al., 2022). Differentiation amongst these interpretations depends upon the experimental paradigm, but variation in methodologies has also made interspecific comparisons difficult (Zeiträg et al., 2022). In any case, gaze following appears to serve different functions across different species, varying form reflexive responses to visual perspective taking; it is therefore species-specific with respect to interpretation across a wide range of taxonomic groups (van Rooijen, 2010; Wilkinson et al., 2010; Leliveld et al., 2013; Davidson and Clayton, 2016).

An additional consideration is that different gaze measures may tap into different cognitive mechanisms. For example, Daum et al. (2012) proposes that a dissociation between infants’ “online” measures such as predictive gaze and *post hoc* measures such as looking time could be due to different processing pathways when viewing goal-directed actions–one pathway processes goal location, the other goal identity. Alternatively, such differences may be due to processing state–predictive gaze relies on incomplete information, in contrast to *post hoc* measures, which respond to a completed action, and have more processing time. Together, such studies highlight that when measuring gaze, care is needed to account for perceptual, contextual, temporal and social variance before drawing interpretations about cognition.

Finally, confounding factors are known to influence experimental results. These may include (a) laterality effects; (b) habituation effects; (c) circadian and other timing effects. For example, in primates including humans, there is a right hemisphere superiority for emotional face processing (e.g., Lindell, 2013) which can result in differential looking patterns toward faces presented in the left versus right-visual-field (Howarth et al., 2021). Hemispheric specialization for visual processing has been demonstrated across invertebrate and vertebrate species (Rogers, 2014), with similarities and differences for emotional information (Leliveld et al., 2013). Laterality effects are therefore widespread and species-specific. Habituation to repeatedly shown stimuli (known as visual adaptation over the short term–seconds, minutes–and learning over longer time periods–hours, days, years) is a well documented neurological response to adapt to changes in the environment. Interest in test stimuli typically declines with repeated presentations, an effect which can be detected in gaze responses over just a few trials (Howarth et al., 2021). Circadian changes in alertness and arousal are also well documented across species, and in captivity these may be further influenced by husbandry procedures (Howarth et al., 2021). Confounds can be controlled for with careful experimental design, and through inclusion as control variables and random effects in statistical analyses.

## Solutions to current limitations

### Experimental validation

Besides careful consideration of data interpretation, we encourage additional steps to improving the use of gaze measures to assess cognition. One option is to improve current methods through assessing measurement consistency. For example, attention in rhesus macaques (*Macaca mulatta*), measured as gaze duration to images of conspecific faces, showed high repeatability across a 4 year period, even when tested using different approaches [automated presentation compared with manually presented images (Howarth et al., 2021)]. Values fell above the average reported for behavioral trait repeatability (R∼0.35) (Bell et al., 2009) and were greater for viewing threat (*R* = 0.63) compared with neutral (*R* = 0.39) faces. Similarly, looking time in 11-month old infants correlated across different screen-based tasks, although not between screen-based and “live” viewing conditions (Wass, 2014). Perez and Feigenson (2021) also report individual repeatability in attention to a violation of expectation condition in infants across a 6 month period (*r* = 0.38). Correlation was not found for infants given an “expected” condition, suggesting that the violation of expectation measure tapped into more than just visual engagement with the stimuli. To address issues of novelty following familiarization trials in the violation of expectation paradigm, Wang (2004) presented 3–4 month old infants with an object permanence test without presenting a prior familiarization task. Infants looked longer in the incongruent (wide object fits behind narrow occluder) than congruent conditions (wide object fits behind wide occluder). In parallel, infants given a control condition comparison did not differentiate their visual attention (Wang, 2004). The author suggests that these findings rule out the possibility that infants’ visual responses are simply a result of habituation during the typical familiarization phase, that is, they are responding to more than just perceptual differences between the conditions.

A second consideration when accounting for gaze is not just the time spent looking toward a stimulus, but also the time spent looking away (Bethell et al., 2012; Rubio-Fernández, 2019). Whilst a subject might not continue to look toward a previously attended stimulus, they could still be processing it whilst looking elsewhere (i.e., the stimulus has captured their attention, neurologically speaking). Visual avoidance of threatening stimuli in humans can be characteristic of extreme anxiety, including phobia (Bögels and Mansell, 2004). More recently, the significance of visual avoidance of threat has been investigated in animals indicating some individuals may be prone to avoidant attentional profiles, especially when stressed (Bethell et al., 2012). Taking a more holistic approach to measuring gaze behavior could thereby provide a more complete picture of the different components of visual attention. Indeed, in studying attention to facial stimuli, averted gaze response should be considered for species who find direct gaze threatening (Coss et al., 2002; Morton et al., 2016).

A third option is to consider behavioral validation of gaze responses. In a task examining canine (*Canis familiaris*) help-seeking, Hirschi et al. (2022) differentiated potentially different meanings of gaze with time spent interacting with a food puzzle: They predicted that if human-directed gaze was an indication of social problem-solving, then it would be correlated with time spent interacting with the box. Alternatively, if gaze indicated giving up, then it would be negatively related to time spent on the box. Results indicated support for the former prediction. Visual engagement with a stimulus does also predict preference in forced choice tasks, for humans viewing facial images (Glaholt and Reingold, 2009) and long-tailed macaques (*Macaca fascicularis*) viewing non-social stimuli (Wilson et al., 2021). Matching visual stimuli with vocal stimuli may also help to identify “recognition” responses via incongruent cross-modal information (Sliwa et al., 2011; Albuquerque et al., 2016). Moreover, gaze responses from experiments can reflect real-world behavior: in 3- and 6-month old rhesus macaques, time spent looking at eye regions of facial images correlated with time spent interacting with peers as well as initiation of social interactions (Ryan et al., 2020).

Alternatively, studies might consider adding methods that do not rely on gaze. Approach latency (Morton et al., 2016; Wathan et al., 2016), frequency (Plimpton et al., 1981), forced choice approach (Cheries et al., 2008; Mascalzoni et al., 2010) and response-slowing tasks (Bethell et al., 2016) provide a simple way to measure response to social stimuli and may more directly reflect real-life decision-making. Forced-choice image discrimination has been trained in horses (*Equus ferus caballus*) (Lansade et al., 2020) and rainbow trout (*Oncorhynchus mykiss*) (Kleiber et al., 2021) as a way to test recognition. Examination of species-specific behavioral and physiological parameters, such as partner-specific displays (Satoh et al., 2016), emotional reactions (Plimpton et al., 1981), sexual receptivity (Clark and Uetz, 1993), changes in ear posture (Bellegarde et al., 2017), or changes in heart rate (Smith et al., 2016; Trösch et al., 2019) also provide informative approaches to understanding context-specific responses, such as recognition or preference. Incorporating such behavioral and physiological measures alongside gaze assessment would help to validate interpretations about visual bias in social contexts.

### Investing in technological solutions

An alternative option in establishing interpretable gaze measures is the use of technological adaptations that allow more in-depth assessment of visual and behavioral responses. Eye tracking is an obvious choice for people working with captive animals, and in recent years advances in infra-red eye tracking have allowed for setups to track gaze in unrestrained primates (Hopper et al., 2020; Lewis and Krupenye, 2022) and trained dogs (Somppi et al., 2012; Kis et al., 2017; Abdai et al., 2021). Gaze studies have even been expanded beyond mammals (Winsor et al., 2021) to include birds (Kjærsgaard et al., 2008; Tyrrell et al., 2014), octopus (*Octopus bimaculoides*) (Taylor, 2020), and jumping spiders (*Phidippus audax*) (Jakob et al., 2018), although currently these methods still usually involve some kind of physical restraint, limiting natural behavioral responses. One consideration is that utilizing eye tracking does not necessarily avoid the issues outlined above, such as ruling out perceptual explanations of gaze responses or identifying whether gaze is motivated by surprise, recognition, preference, or other factors. As with the previously discussed paradigms, eye tracking should utilize rigorous controls and clear linking hypotheses that justify why a given stimulus should elicit a given response (Aslin, 2007). For example, predictions grounded in ecologically relevant behaviors can help to disentangle attentional differences to stimuli, such as images of in-group versus out-group members (Brooks et al., 2022). Accounting for these issues, the benefits of eye tracking over “free viewing” paradigms are multifold.

Firstly, over and above free viewing studies, eye tracking can account for differences in eye movements when viewing a given stimulus. Eye movements can be divided into fixations and saccades. Fixations are when the eyes are fixed on one spot, allowing the viewer to acquire new information, whereas saccades are the movements between fixations; reduction in visual sensitivity means that information intake is restricted during saccades, but information processing still takes place (Irwin, 1998; Rayner, 2009). Notably, the fixation location does not necessarily reflect the point of cognitive focus, that is, a fixation does not necessarily indicate attention (Irwin, 2004). Additionally, eye movements, such as fixation duration and saccade length, also vary greatly between task types (Rayner, 2009). Eye tracking thus allows researchers the flexibility to choose the measures most suited to their research question, as well as task type. Eye tracking studies tend to fall into one of three classes: reflexive orienting, which can examine visual bias; voluntary saccades, used in studies where subjects are directed to look to a given stimulus; and spontaneous scan paths, which examine temporal gaze patterns, such as reading and scene exploration (Eckstein et al., 2017). All of these, in theory, could be used with non-verbal subjects, although voluntary saccades need time to develop in human infants (Eckstein et al., 2017) and a directed approach would require training with non-human animals.

Secondly, eye tracking allows one to assess not just whether a subject attends to one image more than another, but what features within that image are salient for those subjects. Comparative studies have revealed, for example, that when viewing faces, humans tend to fixate longer than non-human apes on the eye regions of faces (Dahl et al., 2009; Kano et al., 2012; Chertoff et al., 2018), and that when viewing goal-directed behavior, humans attend more to the faces of agents than chimpanzees (Myowa-Yamakoshi et al., 2012, 2015). Utilizing an understanding of species-specific viewing patterns could allow one to distinguish between gaze patterns that reflect different cognitive processes. For example, in humans, recognition of a stimulus can be achieved in just two fixations [e.g., Hsiao and Cottrell (2008)]. Exploration patterns (Eckstein et al., 2017; Coutrot et al., 2018) could be applied to account for responses within specific time windows - such as how subjects distribute attention to information within social scenes (Kano and Tomonaga, 2009; Webb et al., 2009; Nakano et al., 2010; McFarland et al., 2013) and identifying anticipatory responses in action sequences (Kano and Call, 2014; Kano and Hirata, 2015; Krupenye et al., 2016).

Finally, this approach offers additional measures beyond gaze location that could be informative for studying cognition. One of these is pupil dilation, which may complement looking time measures (Jackson and Sirois, 2009). Whilst pupil diameter is light sensitive, changes to pupil size under consistent luminance can provide a measure of cognitive load, attention and arousal (Beatty, 1982; Karatekin, 2004; Bradley et al., 2008). In preferential looking time paradigms and violation of expectation studies, measuring pupil size alongside gaze could help researchers to distinguish possible explanations for visual bias. For example, pupil dilation has been found in response to expectation violation in infants (Sirois and Jackson, 2012; Dörrenberg et al., 2018) and to motion violations in dogs (Völter and Huber, 2022), and is indicative of emotional arousal in a helping task in chimpanzees (Hepach et al., 2021). Pupillometry has also been used in chimpanzees to examine pupil-mimicry (Kret et al., 2014), and given varied applications in human research (Eckstein et al., 2017) has the potential to be applied more broadly in cognitive research with non-human subjects. Similar to pupil diameter, microsaccades, which are small saccades produced during gaze fixation (as opposed to saccades, which are produced between gaze fixations) (Martinez-Conde et al., 2013), may provide an indication of cognitive load. For example, when adult humans are instructed to prepare to look away from a target (instead of toward it), they produce fewer microsaccades, and a larger pupil size, although only when presented with mixed trial types (Dalmaso et al., 2020b). Microsaccades can also be informative to gaze orientation, with applications to gaze following studies (Deaner and Platt, 2003). A third, and less explored possibility, is that of blink rate. Blink rate may act as an indirect measure of dopamine activity (Jongkees and Colzato, 2016; Eckstein et al., 2017), a neurotransmitter that is important for “cognitive effort” such as executive function, learning, and decision-making (Westbrook and Braver, 2016). Blink rate is linked to cognitive flexibility in humans (Eckstein et al., 2017), and in Japanese macaques decreases during grooming compared to resting states, suggesting concentration (Hikida, 2022). In rhesus macaques, it has been shown to change in response to presentation of social and emotional information (Ballesta et al., 2016), and increased blink rate is correlated with group size across 71 primate species (Tada et al., 2013). These studies suggest that it may provide a useful measure in the assessment of socio-cognitive traits with non-human primates. Notably, the latter two studies coded blink rate from video footage of faces, indicating that this measure can also be assessed in completely natural settings.

Beyond eye tracking, Artificial Intelligence offers a new set of technological tools to measure gaze from high quality digital images. This would allow high throughput, instantaneously extracting information in near-real-time, and achieving accuracy comparable to human coders (Mathis et al., 2018). Machine Learning algorithms now exist for identifying individuals by their facial characteristics in facially heterogeneous species ranging from birds (Ferreira et al., 2020) to primates (Witham, 2018) and online databases are becoming available [e.g., DeepLabCut Model Zoo: Mathis et al. (2018)]. Within macaque faces, it is possible to landmark facial features such as eyes and pupils (Witham, 2018; Bethell et al., 2022) which, when triangulated with other metrics such as head pose and stimulus location, show promise for assessing direction of gaze with respect to known stimuli (Bethell et al., 2022). It is early days for the application of Machine Learning approaches to eye gaze detection, even in humans where resources are most heavily invested for commercial application (e.g., Khan et al., 2020), and currently initial image gathering, labeling and training is time consuming for each new application. As Machine Learning becomes more widespread there are likely to be substantial developments in the coming years that will improve the efficacy of these approaches for work with animals.

### Collaborative research

Historically, laboratories have aimed to standardize their tests, characteristics of test subjects, and environmental conditions to minimize variability. However, it can be argued that these standardization efforts create results that are vulnerable to site- and population-specific idiosyncrasies and make reproducing them difficult (Würbel, 2000; Voelkl et al., 2018). In contrast, a multi-site approach values data heterogeneity and accounts for intra-specific variation. Consequently, human psychological research was the first to adapt such an approach to improve reproducibility and applicability in their respective research field (e.g., Many Labs, ManyBabies), while initiatives in non-human species have started to follow suit (e.g., ManyPrimates et al., 2019; Lambert et al., 2022; ManyDogs Project et al., 2022). Such multi-lab approaches can also help to facilitate interdisciplinary work between researchers of different fields (e.g., developmental and comparative) to help advance methodological issues. For example, studies examining questions that branch both comparative and development approaches, such as the uncanny valley effect (Mori et al., 2012; Brink et al., 2019; Siebert et al., 2020; Wilson et al., 2020; Carp et al., 2022; Diel et al., 2022) or Theory of Mind (Krupenye et al., 2016; Buttelmann et al., 2017; Dörrenberg et al., 2018), could benefit from more unity between these fields.

## Benefits to research and welfare applications

### Study design and animal welfare

Decision-making tasks are often used to investigate the perceptive and cognitive processes of non-human animals. There are, however, pitfalls: during these tasks, animals often undergo potentially stressful prolonged habituation and training. This can impact individual performance in subsequent experimental tasks via a potential decrease of motivation to participate. For example, tasks that require extensive training may result in self-selected samples where some animals are intrinsically motivated to work or find the tasks enriching, while others might struggle to learn the task contingencies or lack dexterity for manual responses. Prolonged, and potentially stressful, training and isolation can also impact on an individual’s learning performance (Mendl et al., 1997). Moreover, this is also relevant in terms of the implementation of the 3Rs principle as it can severely impact the welfare of the tested animals due to the stress they are experiencing (Refinement).

Assessing gaze as a measure of attention to specific stimuli might be a more beneficial approach, in terms of the ecological validity of the data that will be collected and the animals that are subjected to the tasks. Gaze duration paradigms that utilize biologically relevant stimuli have the advantage that they are not based on excessive training regimes (e.g., used to shape a subject‘s behavior to indicate a choice), anatomical endowments (for example, the presence of hands), or specific motor abilities (e.g., needed in manipulating a device). Assessing gaze has the additional advantage that it does not rely on individually learned behavioral responses (i.e., training), so animals can be repeatedly tested in several similar test series as no carry-over learning bias will occur – additionally improving a better incorporation of the 3Rs principle in animal research (Reduction).

These outlined points might be particularly relevant when we want to assess the cognitive capacities of prey animal species. Prey animals usually pay increased visual attention to potentially dangerous situations. Looking time measures could be used to capture this so-called startle response, a reaction shown by many prey species, such as ungulates, where their attention becomes fixated on novel (and thus potentially threatening) stimuli. With higher alert behavior and, thus, stress levels, prey animals might also benefit from reduced time spent in isolation for habituation and training purposes. Welfare-wise, this approach would be of particular relevance when we want to assess the wellbeing of animals kept under human care - with many of them being prey species (not only in farms, but also in zoos).

### Gaze measures as indicators of welfare

One advantage of using gaze as a behavioral measure is that it may directly reflect individual differences in fundamental aspects of social cognition such as interest, motivation, emotional disposition as well as more subtle aspects such as fearfulness, avoidance and depression. This could be especially useful for identifying animals most “at risk” for compromised welfare, allowing for pre-emptive management interventions. For example, lack of visual attention to events in the social group is a partial diagnostic criteria for depressive-like behavior in primate models of depression (Shively and Willard, 2012). Methods to identify early signs of reduced visual scanning could help alert care staff to individuals suffering within the current environment.

In humans, cognitive models of emotion reveal that visual attention toward biologically relevant stimuli (such as emotional faces) varies with underlying emotion. People who are anxious become overly attentive to threat cues, while phobics may go to extremes to avoid phobia-related stimuli altogether. This well established relationship between emotions like anxiety and attention–measured either as gaze duration or reaction times (e.g., on the dot probe task)–is supported by a large body of literature (Bar-Haim et al., 2007). Similarly, rhesus macaques tested at a 4 year interval showed high repeatability for duration of gaze toward pictures of threatening conspecific faces (Howarth et al., 2021), with a few individuals avoiding threat faces altogether (shortest look duration = 0 ms) and others spending most of the 3 s trial looking toward the threat face (longest look duration = 2,912 ms). Extremes of attention toward (or away from) threat may be associated with underlying anxiety which, if chronically elevated, poses a welfare concern (Bethell et al., 2012). Attention bias tasks have been proposed as a non-invasive tool that could be applied to distinguish acute from chronic anxiety across taxonomic groups (Crump et al., 2018).

Sensitivity to gaze may also be an important consideration for management of animals in captivity, either because prey species may be highly sensitive to direct gaze from humans or because social species are sensitive to the gaze of conspecifics (Davidson and Clayton, 2016). Providing visual barriers that allow animals to avoid direct gaze from both humans and conspecifics is a fundamental enrichment refinement for socially housed and/or prey species where direct gaze may be perceived as a threat (Reinhardt, 2004).

## Conclusion

There are multiple approaches to, and applications of, measuring gaze to study animal and infant cognition. Whilst we caution for better experimental controls and more robust validation of these approaches, we also advocate the use of gaze measures under careful interpretation, proposing solutions that can avoid common pitfalls associated with this methodology. In addition, the use of gaze measurements could be beneficial when accounting for animal welfare, by providing a low-impact approach to testing, and could also be useful as a welfare indicator. Moreover, advances in technology and collaborative research have the potential to open the door for large-scale, robust and non-invasive cognitive testing of a wide range of species.

## Data availability statement

The original contributions presented in this study are included in the article/supplementary material, further inquiries can be directed to the corresponding author.

## Author contributions

VW: conceptualization, writing—original draft preparation, and writing—reviewing and editing. EB and CN: conceptualization and writing—reviewing and editing. All authors contributed to the article and approved the submitted version.

## References

[B1] Abdai J. Ferdinandy B. Lengyel A. Miklósi Á. (2021). Animacy perception in dogs (*Canis familiaris*) and humans (*Homo sapiens*): comparison may be perturbed by inherent differences in looking patterns. *J. Comp. Psychol.* 135 82–88. 10.1037/com0000250 32852965

[B2] Adams L. C. Macdonald S. E. (2018). Spontaneous preference for primate photographs in Sumatran orangutans (*Pongo abelii*). *Int. J. Comp. Psychol.* 31 1–16. 10.46867/ijcp.2018.31.04.05

[B3] Albuquerque N. Guo K. Wilkinson A. Savalli C. Otta E. Mills D. (2016). Dogs recognize dog and human emotions. *Biol. Lett.* 12:20150883. 10.1098/rsbl.2015.0883 26763220 PMC4785927

[B4] Arre A. M. Clark C. S. Santos L. R. (2020). Do young rhesus macaques know what others see? A comparative developmental perspective. *Am. J. Primatol.* 82:e23054. 10.1002/ajp.23054 31566777 PMC7103490

[B5] Aslin R. N. (2007). What’s in a look? *Dev. Sci.* 10 48–53. 10.1111/j.1467-7687.2007.00563.x 17181699 PMC2493049

[B6] Baciadonna L. Briefer E. F. Favaro L. McElligott A. G. (2019). Goats distinguish between positive and negative emotion-linked vocalisations. *Front. Zool.* 16:25. 10.1186/s12983-019-0323-z 31320917 PMC6617626

[B7] Baillargeon R. Hanko-Summers S. (1990). Is the top object adequately supported by the bottom object? Young infants’ understanding of support relation. *Cogn. Dev.* 5 29–53.

[B8] Ballesta S. Mosher C. P. Szep J. Fischl K. D. Gothard K. M. (2016). Social determinants of eyeblinks in adult male macaques. *Sci. Rep.* 6:38686. 10.1038/srep38686 27922101 PMC5138631

[B9] Bar-Haim Y. Lamy D. Pergamin L. Bakermans-Kranenburg M. J. van IJzendoorn M. H. (2007). Threat-related attentional bias in anxious and nonanxious individuals: a meta-analytic study. *Psychol. Bull.* 133 1–24. 10.1037/0033-2909.133.1.1 17201568

[B10] Beatty J. (1982). Task-evoked pupillary responses, processing load, and the structure of processing resources. *Psychol. Bull.* 91 276–292. 7071262

[B11] Bell A. M. Hankison S. J. Laskowski K. L. (2009). The repeatability of behaviour: a meta-analysis. *Anim. Behav.* 77 771–783. 10.1016/j.anbehav.2008.12.022 24707058 PMC3972767

[B12] Bellegarde L. G. A. Haskell M. J. Duvaux-ponter C. Weiss A. Boissy A. Erhard H. W. (2017). Face-based perception of emotions in dairy goats. *Appl. Anim. Behav. Sci.* 193 51–59. 10.1016/j.applanim.2017.03.014

[B13] Bergman T. J. Beehner J. C. Cheney D. L. Seyfarth R. M. (2003). Hierarchical classification by rank and kinship in baboons. *Science* 302 1234–1236. 10.1126/science.1087513 14615544

[B14] Bethell E. J. Holmes A. MacLarnon A. Semple S. (2012). Evidence that emotion mediates social attention in rhesus macaques. *PLoS One* 7:e44387. 10.1371/journal.pone.0044387 22952968 PMC3431396

[B15] Bethell E. J. Holmes A. Maclarnon A. Semple S. (2016). Emotion evaluation and response slowing in a non-human primate: new directions for cognitive bias measures of animal emotion? *Behav. Sci.* 6 1–16. 10.3390/bs6010002 26761035 PMC4810036

[B16] Bethell E. J. Khan W. Hussain A. (2022). A deep transfer learning model for head pose estimation in rhesus macaques during cognitive tasks: towards a nonrestraint noninvasive 3Rs approach. *Appl. Anim. Behav. Sci.* 255:105708. 10.1016/j.applanim.2022.105708

[B17] Bogartz R. S. Shinskey J. L. Schilling T. H. (2000). Object permanence in five-and-a-half-month-old infants? *Infancy* 1 403–428. 10.1207/S15327078IN0104_3 32680304

[B18] Bögels S. M. Mansell W. (2004). Attention processes in the maintenance and treatment of social phobia: hypervigilance, avoidance and self-focused attention. *Clin. Psychol. Rev.* 24 827–856. 10.1016/j.cpr.2004.06.005 15501558

[B19] Bradley M. M. Miccoli L. Escrig M. A. Lang P. J. (2008). The pupil as a measure of emotional arousal and autonomic activation. *Psychophysiology* 45 602–607. 10.1111/j.1469-8986.2008.00654.x 18282202 PMC3612940

[B20] Brink K. A. Gray K. Wellman H. M. (2019). Creepiness creeps in: uncanny valley feelings are acquired in childhood. *Child Dev.* 90 1202–1214. 10.1111/cdev.12999 29236300

[B21] Brooks J. Kano F. Kawaguchi Y. Yamamoto S. (2022). Oxytocin promotes species-relevant outgroup attention in bonobos and chimpanzees. *Horm. Behav.* 143:105182. 10.1016/j.yhbeh.2022.105182 35537292

[B22] Buttelmann D. Buttelmann F. Carpenter M. Call J. Tomasello M. (2017). Great apes distinguish true from false beliefs in an interactive helping task. *PLoS One* 12:e0173793. 10.1371/journal.pone.0173793 28379987 PMC5381863

[B23] Cacchione T. Krist H. (2004). Recognizing impossible object relations: intuitions about support in chimpanzees (*Pan troglodytes*). *J. Comp. Psychol.* 118 140–148. 10.1037/0735-7036.118.2.140 15250801

[B24] Cannon E. N. Woodward A. L. (2012). Infants generate goal-based action predictions. *Dev. Sci.* 15 292–298. 10.1111/j.1467-7687.2011.01127.x.Infants 22356184 PMC3612028

[B25] Carp S. B. Santistevan A. C. Machado C. J. Whitaker A. M. Aguilar B. L. Bliss-Moreau E. (2022). Monkey visual attention does not fall into the uncanny valley. *Sci. Rep.* 12:11760. 10.1038/s41598-022-14615-x 35817791 PMC9273626

[B26] Charlton B. D. Ellis W. A. H. Larkin R. Tecumseh Fitch W. (2012). Perception of size-related formant information in male koalas (*Phascolarctos cinereus*). *Anim. Cogn.* 15 999–1006. 10.1007/s10071-012-0527-5 22740017

[B27] Cheney D. L. Seyfarth R. M. Silk J. B. (1995). The responses of female baboons (*Papio cynocephalus ursinus*) to anomalous social interactions: evidence for causal reasoning? *J. Comp. Psychol.* 109 134–141. 10.1037/0735-7036.109.2.134 7758289

[B28] Cheries E. W. Mitroff S. R. Wynn K. Scholl B. J. (2008). Cohesion as a principle of object persistence in infancy. *Dev. Sci.* 11 427–432. 10.1111/j.1467-7687.2008.00687.x 18466376 PMC3665331

[B29] Chertoff S. Margulis S. Rodgers J. D. (2018). Visual processing of faces in juvenile western lowland gorillas without the use of training or reinforcement: a pilot study. *Anim. Behav. Cogn.* 5 292–299. 10.26451/abc.05.03.04.2018

[B30] Clark D. L. Uetz G. W. (1993). Signal efficacy and the evolution of male dimorphism in the jumping spider, *Maevia inclemens*. *Proc. Natl. Acad. Sci. U.S.A.* 90 11954–11957. 10.1073/pnas.90.24.11954 11607446 PMC48103

[B31] O’Connell S. Dunbar R. I. M. (2005). The perception of causality in chimpanzees (*Pan spp.*). *Anim. Cogn.* 8 60–66. 10.1007/s10071-004-0231-1 15322943

[B32] Coss R. G. Marks S. Ramakrishnan U. (2002). Early environment shapes the development of gaze aversion by wild bonnet macaques (*Macaca radiata*). *Primates* 43 217–222. 10.1007/BF02629649 12145402

[B33] Coutrot A. Hsiao J. H. Chan A. B. (2018). Scanpath modeling and classification with hidden Markov models. *Behav. Res. Methods* 50 362–379. 10.3758/s13428-017-0876-8 28409487 PMC5809577

[B34] Craighero L. Leo I. Umiltà C. Simion F. (2011). Newborns’ preference for goal-directed actions. *Cognition* 120 26–32. 10.1016/j.cognition.2011.02.011 21388616

[B35] Crump A. Arnott G. Bethell E. J. (2018). Affect-driven attention biases as animal welfare indicators: review and methods. *Animals* 8:136. 10.3390/ani8080136 30087230 PMC6115853

[B36] Dahl C. D. Wallraven C. Bülthoff H. H. Logothetis N. K. (2009). Humans and macaques employ similar face-processing strategies. *Curr. Biol.* 19 509–513. 10.1016/j.cub.2009.01.061 19249210

[B37] Dalmaso M. Castelli L. Galfano G. (2020a). Social modulators of gaze-mediated orienting of attention: a review. *Psychon. Bull. Rev.* 27 833–855. 10.3758/s13423-020-01730-x 32291650

[B38] Dalmaso M. Castelli L. Galfano G. (2020b). Microsaccadic rate and pupil size dynamics in pro-/anti-saccade preparation: the impact of intermixed vs. blocked trial administration. *Psychol. Res.* 84 1320–1332. 10.1007/s00426-018-01141-7 30603866

[B39] Dasser V. Ulbaek I. Premack D. (1989). The perception of intention. *Science* 243 365–367. 10.1126/science.2911746 2911746

[B40] Daum M. M. Attig M. Gunawan R. Prinz W. Gredebäck G. (2012). Actions seen through babies’ eyes: a dissociation between looking time and predictive gaze. *Front. Psychol.* 3:370. 10.3389/fpsyg.2012.00370 23060838 PMC3459021

[B41] Davidson G. L. Clayton N. S. (2016). New perspectives in gaze sensitivity research. *Learn. Behav.* 44 9–17. 10.3758/s13420-015-0204-z 26582567

[B42] Deaner R. O. Platt M. L. (2003). Reflexive social attention in monkeys and humans. *Curr. Biol.* 13 1609–1613. 10.1016/j.cub.2003.08.025 13678591

[B43] Diel A. Weigelt S. Macdorman K. F. (2022). A meta-analysis of the uncanny valley’s independent and dependent variables. *ACM Trans. Hum. Robot Interact.* 11 1–33. 10.1145/3470742

[B44] Dörrenberg S. Rakoczy H. Liszkowski U. (2018). How (not) to measure infant Theory of Mind: testing the replicability and validity of four non-verbal measures. *Cogn. Dev.* 46 12–30. 10.1016/j.cogdev.2018.01.001 33204438

[B45] Dufour V. Pascalis O. Petit O. (2006). Face processing limitation to own species in primates, a response to social needs? *Behav. Processes* 73 107–113. 10.1016/j.beproc.2006.04.006 16690230

[B46] Eckstein M. K. Guerra-Carrillo B. Miller Singley A. T. Bunge S. A. (2017). Beyond eye gaze: what else can eyetracking reveal about cognition and cognitive development? *Dev. Cogn. Neurosci.* 25 69–91. 10.1016/j.dcn.2016.11.001 27908561 PMC6987826

[B47] Elsner B. Adam M. (2021). Infants’ goal prediction for simple action events: the role of experience and agency cues. *Top. Cogn. Sci.* 13 45–62. 10.1111/tops.12494 32128981

[B48] Fantz R. L. (1958a). Visual discrimination in a neonate chimpanzee. *Percept. Mot. Skills* 8 59–66.

[B49] Fantz R. L. (1958b). Pattern vision in young infants. *Psychol. Rec.* 8 43–47.

[B50] Ferreira A. C. Silva L. R. Renna F. Brandl H. B. Renoult J. P. Farine D. R. (2020). Deep learning-based methods for individual recognition in small birds. *Methods Ecol. Evol.* 11 1072–1085. 10.1111/2041-210X.13436

[B51] Franchini M. Glaser B. de Wilde H. W. Gentaz E. Eliez S. Schaer M. (2017). Social orienting and joint attention in preschoolers with autism spectrum disorders. *PLoS One* 12:e0178859. 10.1371/journal.pone.0178859 28599002 PMC5466314

[B52] Fujita K. (1987). Species recognition by five macaque monkeys. *Primates* 28 353–366.

[B53] Glaholt M. G. Reingold E. M. (2009). Predicting preference from fixations. *PsychNology J.* 7 141–158. 10.1037/e527342012-455

[B54] Gothard K. M. Erickson C. A. Amaral D. G. (2004). How do rhesus monkeys (*Macaca mulatta*) scan faces in a visual paired comparison task?? *Anim. Cogn.* 7 25–36. 10.1007/s10071-003-0179-6 14745584

[B55] Haith M. M. (1998). Who put the cog in infant cognition? Is rich interpretation too costly? *Infant Behav. Dev.* 21 167–179. 10.1016/S0163-6383(98)90001-7

[B56] Hayashi T. Akikawa R. Kawasaki K. Egawa J. Minamimoto T. Koboyashi K. (2020). Macaques exhibit implicit gaze bias anticipating others’ false-belief-driven actions via medial prefrontal cortex article. *Cell Rep.* 30 4433–4444. 10.1016/j.celrep.2020.03.013 32234478

[B57] Hepach R. Vaish A. Kano F. Albiach-Serrano A. Benziad L. Call J. (2021). Chimpanzees’ (*Pan troglodytes*) internal arousal remains elevated if they cannot themselves help a conspecific. *J. Comp. Psychol.* 135 196–207. 10.1037/com0000255 33315411

[B58] Hikida K. (2022). Eyeblink rate as an indicator of concentration on grooming in Japanese macaques (*Macaca fuscata*). *Am. J. Primatol.* 84:e23392. 10.1002/ajp.23392 35612538

[B59] Hirschi A. Mazzini A. Riemer S. (2022). Disentangling help-seeking and giving up: differential human-directed gazing by dogs in a modified unsolvable task paradigm. *Anim. Cogn.* 25 821–836. 10.1007/s10071-021-01595-0 35020108 PMC8753593

[B60] Hopper L. M. Gulli R. A. Howard L. H. Kano F. Krupenye C. Ryan A. M. (2020). The application of noninvasive, restraint-free eye-tracking methods for use with nonhuman primates. *Behav. Res. Methods* 53 1003–1030. 10.3758/s13428-020-01465-6 32935327

[B61] Howard L. H. Festa C. Lonsdorf E. V. College M. (2018). Through their eyes: the influence of social models on attention and memory in capuchin monkeys (*Sapajus apella*). *J. Comp. Psychol.* 132 210–219. 10.1037/com0000111 29517249

[B62] Howarth E. R. I. Kemp C. Thatcher H. R. Szott I. D. Farningham D. Witham C. L. (2021). Developing and validating attention bias tools for assessing trait and state affect in animals: a worked example with *Macaca mulatta*. *Appl. Anim. Behav. Sci.* 234:105198. 10.1016/j.applanim.2020.105198

[B63] Hsiao J. H. Cottrell G. (2008). Two fixations suffice in face recognition. *Psychol. Sci.* 19 998–1006. 10.1111/j.1467-9280.2008.02191.x 19000210 PMC7360057

[B64] Irwin D. E. (1998). Lexical processing during saccadic eye movements. *Cognit. Psychol.* 36 1–27. 9679075 10.1006/cogp.1998.0682

[B65] Irwin D. E. (2004). “Fixation location and fixation duration as indices of cognitive processing,” in *The interface of language, vision and action: eye movements and the visual world*, eds Henderson J. M. Ferreira F. (New York, NY: Psychology Press), 105–134.

[B66] Jackson I. Sirois S. (2009). Infant cognition: going full factorial with pupil dilation. *Dev. Sci.* 12 670–679. 10.1111/j.1467-7687.2008.00805.x 19635092

[B67] Jakob E. M. Long S. M. Harland D. P. Jackson R. R. Carey A. Searles M. E. (2018). Lateral eyes direct principal eyes as jumping spiders track objects. *Curr. Biol.* 28 R1092–R1093. 10.1016/j.cub.2018.07.065 30253146

[B68] Jongkees B. J. Colzato L. S. (2016). Spontaneous eye blink rate as predictor of dopamine-related cognitive function—A review. *Neurosci. Biobehav. Rev.* 71 58–82. 10.1016/j.neubiorev.2016.08.020 27555290

[B69] Kampis D. Kármán P. Csibra G. Southgate V. Hernik M. (2021). A two-lab direct replication attempt of Southgate, Senju and Csibra (2007). *R. Soc. Open Sci.* 8:210190. 10.1098/rsos.210190 34457336 PMC8386515

[B70] Kano F. Call J. (2014). Great apes generate goal-based action predictions: an eye-tracking study. *Psychol. Sci.* 25 1691–1698. 10.1177/0956797614536402 25022278

[B71] Kano F. Hirata S. (2015). Great apes make anticipatory looks based on long-term memory of single events. *Curr. Biol.* 25 2513–2517. 10.1016/j.cub.2015.08.004 26387711

[B72] Kano F. Tomonaga M. (2009). How chimpanzees look at pictures: a comparative eye-tracking study. *Proc. Biol. Sci.* 276 1949–1955. 10.1098/rspb.2008.1811 19324790 PMC2677242

[B73] Kano F. Call J. Tomonaga M. (2012). Face and eye scanning in gorillas (*Gorilla gorilla*), orangutans (*Pongo abelii*), and humans (*Homo sapiens*): unique eye-viewing patterns in humans among hominids. *J. Comp. Psychol.* 126 388–398. 10.1037/a0029615 22946925

[B74] Karatekin C. (2004). Development of attentional allocation in the dual task paradigm. *Int. J. Psychophysiol.* 52 7–21. 10.1016/j.ijpsycho.2003.12.002 15003369

[B75] Khan W. Hussain A. Kuru K. Al-askar H. (2020). Pupil localisation and eye centre estimation using machine learning and computer vision. *Sensors* 20:3785. 10.3390/s20133785 32640589 PMC7374404

[B76] Kis A. Hernádi A. Miklósi B. Kanizsár O. Topál J. (2017). The way dogs (*canis familiaris*) look at human emotional faces is modulated by oxytocin. An eye-tracking study. *Front. Behav. Neurosci.* 11:210. 10.3389/fnbeh.2017.00210 29163082 PMC5671652

[B77] Kjærsgaard A. Pertoldi C. Loeschcke V. Witzner H. D. (2008). Tracking the gaze of birds. *J. Avian Biol.* 39 466–469. 10.1111/j.2008.0908-8857.04288.x

[B78] Kleiber A. Valotaire C. Patinote A. Sudan P.-L. Gourmelen G. Duret C. (2021). Rainbow trout discriminate 2-D photographs of conspecifics from distracting stimuli using an innovative operant conditioning device. *Learn. Behav.* 49 292–306. 10.3758/s13420-020-00453-2 33409895

[B79] Koba R. Izumi A. (2008). Japanese monkeys (*Macaca fuscata*) discriminate between pictures of conspecific males and females without specific training. *Behav. Processes* 79 70–73. 10.1016/j.beproc.2008.04.005 18515019

[B80] Kret M. E. Tomonaga M. Matsuzawa T. (2014). Chimpanzees and humans mimic pupil-size of conspecifics. *PLoS One* 9:e104886. 10.1371/journal.pone.0104886 25140998 PMC4139319

[B81] Krupenye C. Kano F. Hirata S. Call J. Tomasello M. (2016). Great apes anticipate that other individuals will act according to false beliefs. *Science* 354 110–114. 10.1126/science.aaf8110 27846501

[B82] Lambert M. Farrar B. Garcia-Pelegrin E. Reber S. Miller R. (2022). ManyBirds: a multi-site collaborative Open Science approach to avian cognition and behavior research. *Anim. Behav. Cogn.* 9 133–152. 10.26451/abc.09.01.11.2022

[B83] Lansade L. Colson V. Parias C. Trösch M. Reigner F. Calandreau L. (2020). Female horses spontaneously identify a photograph of their keeper, last seen six months previously. *Sci. Rep.* 10:6302. 10.1038/s41598-020-62940-w 32286345 PMC7156667

[B84] Leliveld L. M. C. Langbein J. Puppe B. (2013). The emergence of emotional lateralization: evidence in non-human vertebrates and implications for farm animals. *Appl. Anim. Behav. Sci.* 145 1–14. 10.1016/j.applanim.2013.02.002

[B85] Leslie A. M. (1982). The perception of causality in infants. *Perception* 11 173–186. 7155770 10.1068/p110173

[B86] Lewis L. S. Krupenye C. (2022). Eye-tracking as a window into primate social cognition. *Am. J. Primatol.* 84:e23393. 10.1002/ajp.23393 35635515

[B87] Lindell A. (2013). Continuities in emotion lateralization in human and non-human primates. *Front. Hum*. *Neurosci*. 7. 10.3389/fnhum.2013.00464 23964230 PMC3737467

[B88] ManyDogs Project, Alberghina D. Bray E. E. Buchsbaum D. Byosiere S.-E. Espinosa J. (2022). ManyDogs project: a big team science approach to investigating canine behavior and cognition. *PsyArxiv* [preprint] 10.31234/osf.io/j82uc

[B89] ManyPrimates, Altschul D. M. Beran M. J. Bohn M. Call J. DeTroy S. (2019). Establishing an infrastructure for collaboration in primate cognition research. *PLoS One* 14:e0223675. 10.1371/journal.pone.0223675 31648222 PMC6812783

[B90] Martinez-Conde S. Otero-Millan J. Macknik S. L. (2013). The impact of microsaccades on vision: towards a unified theory of saccadic function. *Nat. Rev. Neurosci.* 14 83–96. 10.1038/nrn3405 23329159

[B91] Mascalzoni E. Regolin L. Vallortigara G. (2010). Innate sensitivity for self-propelled causal agency in newly hatched chicks. *Proc. Natl. Acad. Sci. U.S.A.* 107 4483–4485. 10.1073/pnas.0908792107 20160095 PMC2840119

[B92] Mathis A. Mamidanna P. Cury K. M. Abe T. Murthy V. N. Mathis M. W. (2018). DeepLabCut: markerless pose estimation of user-defined body parts with deep learning. *Nat. Neurosci.* 21 1281–1289. 10.1038/s41593-018-0209-y 30127430

[B93] McFarland R. Roebuck H. Yan Y. Majolo B. Li W. Guo K. (2013). Social interactions through the eyes of macaques and humans. *PLoS One* 8:e56437. 10.1371/journal.pone.0056437 23457569 PMC3574082

[B94] Méary D. Wu L. Zhinan L. Guo K. Pascalis O. (2014). Seeing two faces together: preference formation in humans and rhesus macaques. *Anim. Cogn.* 17 1107–1119. 10.1007/s10071-014-0742-3 24638876

[B95] Mendl M. Laughlin K. Hitchcock D. (1997). Pigs in space: spatial memory and its susceptibility to interference. *Anim. Behav.* 54 1491–1508. 10.1006/anbe.1997.0564 9794774

[B96] Mori M. MacDorman K. Kageki N. (2012). The uncanny valley [from the field]. *IEEE Robot. Autom. Mag.* 19 98–100. 10.1109/MRA.2012.2192811

[B97] Morton F. B. Brosnan S. F. Prétôt L. Buchanan-Smith H. M. O’Sullivan E. Stocker M. (2016). Using photographs to study animal social cognition and behaviour: do capuchins’ responses to photos reflect reality? *Behav. Processes* 124 38–46. 10.1016/j.beproc.2015.10.005 26476153

[B98] Myowa-Yamakoshi M. Scola C. Hirata S. (2012). Humans and chimpanzees attend differently to goal-directed actions. *Nat. Commun.* 3:693. 10.1038/ncomms1695 22353723

[B99] Myowa-Yamakoshi M. Yoshida C. Hirata S. (2015). Humans but not chimpanzees vary face-scanning patterns depending on contexts during action observation. *PLoS One* 10:e0139989. 10.1371/journal.pone.0139989 26535901 PMC4633149

[B100] Nakano T. Tanaka K. Endo Y. Yamane Y. Yamamoto T. Nakano Y. (2010). Atypical gaze patterns in children and adults with autism spectrum disorders dissociated from developmental changes in gaze behaviour. *Proc. R. Soc. B* 277 2935–2943. 10.1098/rspb.2010.0587 20484237 PMC2982027

[B101] Onishi K. H. Baillargeon R. (2005). Do 15-month-old infants understand false beliefs? *Science* 308 255–258. 10.1126/science.1107621 15821091 PMC3357322

[B102] Overduin-de Vries A. M. Bakker F. A. A. Spruijt B. M. Sterck E. H. M. (2016). Male long-tailed macaques (*Macaca fascicularis*) understand the target of facial threat. *Am. J. Primatol.* 78 720–730. 10.1002/ajp.22536 26872303

[B103] Paukner A. Wooddell L. J. Lefevre C. E. Lonsdorf E. Lonsdorf E. (2017). Do capuchin monkeys (*Sapajus apella*) prefer symmetrical face shapes? *J. Comp. Psychol.* 131:73. 10.1037/com0000052 28182489 PMC5301575

[B104] Paul E. S. Harding E. J. Mendl M. (2005). Measuring emotional processes in animals: the utility of a cognitive approach. *Neurosci. Biobehav. Rev.* 29 469–491. 10.1016/j.neubiorev.2005.01.002 15820551

[B105] Perez J. Feigenson L. (2021). Stable individual differences in infants’ responses to violations of intuitive physics. *Proc. Natl. Acad. Sci. U.S.A.* 118:e2103805118. 10.1073/pnas.2103805118 34183399 PMC8271639

[B106] Pfefferle D. Kazem A. J. N. Brockhausen R. R. Ruiz-lambides A. V. Widdig A. (2014). Monkeys spontaneously discriminate their unfamiliar paternal kin under natural conditions using facial cues. *Curr. Biol.* 24 1806–1810. 10.1016/j.cub.2014.06.058 25065751 PMC4137972

[B107] Plimpton E. H. Swartz K. B. Rosenblum L. A. (1981). Responses of juvenile bonnet macaques to social stimuli presented through color videotapes. *Dev. Psychobiol.* 14 109–115. 10.1002/dev.420140204 7202848

[B108] Rakotonirina H. Kappeler P. M. Fichtel C. (2018). The role of facial pattern variation for species recognition in red-fronted lemurs (*Eulemur rufifrons*). *BMC Evol. Biol.* 18:19. 10.1186/s12862-018-1126-0 29433448 PMC5809826

[B109] Rayner K. (2009). The 35th Sir Frederick Bartlett Lecture: eye movements and attention in reading, scene perception, and visual search. *Q. J. Exp. Psychol.* 62 1457–1506. 10.1080/17470210902816461 19449261

[B110] Reinhardt V. (2004). Common husbandry-related variables in biomedical research with animals. *Lab. Anim.* 38 213–235. 10.1258/002367704323133600 15207033

[B111] Rendall D. Rodman P. S. Emond R. E. (1996). Vocal recognition of individuals and kin in free-ranging rhesus monkeys. *Anim. Behav.* 51 1007–1015. 10.1006/anbe.1996.0103

[B112] Rogers L. J. (2014). Asymmetry of brain and behavior in animals: its development, function, and human relevance: laterality development in animal models. *Genesis* 52 555–571. 10.1002/dvg.22741 24408478

[B113] Rohr C. R. V. Schaik C. P. V. Kissling A. Burkart J. M. (2015). Chimpanzees’ bystander reactions to infanticide an evolutionary precursor of social norms? *Hum. Nat.* 26 143–160. 10.1007/s12110-015-9228-5 26108616

[B114] Rubio-Fernández P. (2019). Publication standards in infancy research: three ways to make violation-of-expectation studies more reliable. *Infant Behav. Dev.* 54 177–188. 10.1016/j.infbeh.2018.09.009 30814033

[B115] Ryan A. M. Murai T. Lau A. R. Hogrefe C. E. McAllister A. K. Carter C. S. (2020). New approaches to quantify social development in rhesus macaques (*Macaca mulatta*): integrating eye tracking with traditional assessments of social behavior. *Dev. Psychobiol.* 62 950–962. 10.1002/dev.22003 32666534 PMC8754470

[B116] Satoh S. Tanaka H. Kohda M. (2016). Facial recognition in a Discus fish (*Cichlidae*): experimental approach using digital models. *PLoS One* 11:e0154543. 10.1371/journal.pone.0154543 27191162 PMC4871422

[B117] Schell A. Rieck K. Schell K. Hammerschmidt K. Fischer J. (2011). Adult but not juvenile Barbary macaques spontaneously recognize group members from pictures. *Anim. Cogn.* 14 503–509. 10.1007/s10071-011-0383-8 21318387 PMC3117280

[B118] Schloegl C. Kotrschal K. Bugnyar T. (2007). Gaze following in common ravens, *Corvus corax*: ontogeny and habituation. *Anim. Behav.* 74 769–778. 10.1016/j.anbehav.2006.08.017

[B119] Shepherd S. V. (2010). Following gaze: gaze-following behavior as a window into social cognition. *Front. Integr. Neurosci.* 4:5. 10.3389/fnint.2010.00005 20428494 PMC2859805

[B120] Shively C. A. Willard S. L. (2012). Behavioral and neurobiological characteristics of social stress versus depression in nonhuman primates. *Exp. Neurol.* 233 87–94. 10.1016/j.expneurol.2011.09.026 21983263 PMC4031682

[B121] Siebert R. Taubert N. Spadacenta S. Dicke P. W. Giese M. A. Thier P. (2020). A naturalistic dynamic monkey head avatar elicits species-typical reactions and overcomes the uncanny valley. *Eneuro* 7:ENEURO.0524-19.2020. 10.1523/ENEURO.0524-19.2020 32513660 PMC7340843

[B122] Sirois S. Jackson I. R. (2012). Pupil dilation and object permanence in infants. *Infancy* 17 61–78. 10.1111/j.1532-7078.2011.00096.x 32693502

[B123] Sliwa J. Duhamel J.-R. Pascalis O. Wirth S. (2011). Spontaneous voice–face identity matching by rhesus monkeys for familiar conspecifics and humans. *Proc. Natl. Acad. Sci. U.S.A.* 108 1735–1740. 10.1073/pnas.1008169108 21220340 PMC3029706

[B124] Slocombe K. E. Kaller T. Call J. Zuberbuehler K. (2010). Chimpanzees extract social information from agonistic screams. *PLoS One* 5:e11473. 10.1371/Citation PMC2904366 20644722

[B125] Smith A. V. Proops L. Grounds K. Wathan J. McComb K. (2016). Functionally relevant responses to human facial expressions of emotion in the domestic horse (*Equus caballus*). *Biol. Lett.* 12:20150907. 10.1098/rsbl.2015.0907 26864784 PMC4780548

[B126] Somppi S. Törnqvist H. Hänninen L. Krause C. Vainio O. (2012). Dogs do look at images: eye tracking in canine cognition research. *Anim. Cogn.* 15 163–174. 10.1007/s10071-011-0442-1 21861109

[B127] Southgate V. Senju A. Csibra G. (2007). Action anticipation through attribution of false belief by 2 year olds. *Psychol. Sci.* 18 587–592. 10.1111/j.1467-9280.2007.01944.x 17614866

[B128] Spelke E. S. Katz G. Purcell S. E. Ehrlich S. M. Breinlinger K. (1994). Early knowledge of object motion: continuity and inertia. *Cognition* 51 131–176. 10.1016/0010-0277(94)90013-2 8168357

[B129] Tada H. Omori Y. Hirokawa K. Ohira H. Tomonaga M. (2013). Eye-blink behaviors in 71 species of primates. *PLoS One* 8:e66018. 10.1371/journal.pone.0066018 23741522 PMC3669291

[B130] Tafreshi D. Thompson J. J. Racine T. P. (2014). An analysis of the conceptual foundations of the infant preferential looking paradigm. *Hum. Dev.* 57 222–240. 10.1159/000363487

[B131] Taylor M. A. (2020). *Autonomous eye tracking in octopus bimaculoides.* B.Sc. thesis. Hanover, NH: Dartmouth College.

[B132] Trösch M. Cuzol F. Parias C. Calandreau L. Nowak R. Lansade L. (2019). Horses categorize human emotions cross-modally based on facial expression and non-verbal vocalizations. *Animals* 9:862. 10.3390/ani9110862 31653088 PMC6912773

[B133] Tyrrell L. P. Butler S. R. Yorzinski J. L. Fernández-Juricic E. (2014). A novel system for bi-ocular eye-tracking in vertebrates with laterally placed eyes. *Methods Ecol. Evol.* 5 1070–1077. 10.1111/2041-210X.12249

[B134] van Rooijen J. (2010). Do dogs and bees possess a ‘theory of mind’? *Anim. Behav.* 79 e7–e8. 10.1016/j.anbehav.2009.11.016

[B135] Voelkl B. Vogt L. Sena E. S. Würbel H. (2018). Reproducibility of preclinical animal research improves with heterogeneity of study samples. *PLoS Biol*. 16:e2003693. 10.1371/journal.pbio.2003693 29470495 PMC5823461

[B136] Völter C. J. Huber L. (2022). Pupil size changes reveal dogs’ sensitivity to motion cues. *iScience* 25:104801. 10.1016/j.isci.2022.104801 36051183 PMC9424576

[B137] Wang S. (2004). Young infants’ reasoning about hidden objects: evidence from violation-of-expectation tasks with test trials only. *Cognition* 93 167–198. 10.1016/j.cognition.2003.09.012 15178376 PMC4215956

[B138] Wass S. V. (2014). Comparing methods for measuring peak look duration: are individual differences observed on screen-based tasks also found in more ecologically valid contexts? *Infant Behav. Dev.* 37 315–325. 10.1016/j.infbeh.2014.04.007 24905901 PMC4103485

[B139] Wathan J. Proops L. Grounds K. Mccomb K. (2016). Horses discriminate between facial expressions of conspecifics. *Sci. Rep.* 6 1–11. 10.1038/srep38322 27995958 PMC5171796

[B140] Webb A. Knott A. Macaskill M. R. (2009). Eye movements during transitive action observation have sequential structure. *Acta Psychol. (Amst.)* 133 51–56. 10.1016/j.actpsy.2009.09.001 19854431

[B141] Westbrook A. Braver T. S. (2016). Dopamine does double duty in motivating cognitive effort. *Neuron* 89 695–710. 10.1016/j.neuron.2015.12.029 26889810 PMC4759499

[B142] Wilkinson A. Mandl I. Bugnyar T. Huber L. (2010). Gaze following in the red-footed tortoise (*Geochelone carbonaria*). *Anim. Cogn.* 13 765–769. 10.1007/s10071-010-0320-2 20411292

[B143] Wilson V. A. D. Kade C. Fischer J. (2021). Testing the relationship between looking time and choice preference in long-tailed macaques. *Anim. Behav. Cogn.* 8 351–375. 10.26451/abc.08.03.03.2021

[B144] Wilson V. A. D. Kade C. Moeller S. Treue S. Kagan I. Fischer J. (2020). Macaque gaze responses to the Primatar: a virtual macaque head for social cognition research. *Front. Psychol.* 11:1645. 10.3389/fpsyg.2020.01645 32765373 PMC7379899

[B145] Winsor A. M. Pagoti G. F. Daye D. J. Cheries E. W. Cave K. R. Jakob E. M. (2021). What gaze direction can tell us about cognitive processes in invertebrates. *Biochem. Biophys. Res. Commun.* 564 43–54. 10.1016/j.bbrc.2020.12.001 33413978

[B146] Winters S. Dubuc C. Higham J. P. (2015). Perspectives: the looking time experimental paradigm in studies of animal visual perception and cognition. *Ethology* 121 625–640. 10.1111/eth.12378

[B147] Witham C. L. (2018). Automated face recognition of rhesus macaques. *J. Neurosci. Methods* 300 157–165. 10.1016/j.jneumeth.2017.07.020 28739161 PMC5909037

[B148] Würbel H. (2000). Behaviour and the standardization fallacy. *Nat. Genet*. 26:263. 10.1038/81541 11062457

[B149] Zeiträg C. Jensen T. R. Osvath M. (2022). Gaze following: a socio-cognitive skill rooted in deep time. *Front. Psychol.* 13:950935. 10.3389/fpsyg.2022.950935 36533020 PMC9756811

